# Analysis of the Expression Patterns of piRNAs in Response to Microsporidian Invasion in Midgut of Workers (*Apis cerana cerana*)

**DOI:** 10.3390/ijms26062402

**Published:** 2025-03-07

**Authors:** Yiqiong Zhang, Mengyi Wang, Wenhua Xu, He Zang, Tizhen Yan, Tao Wu, Kaifei Huang, Dafu Chen, Qingming Luo, Rui Guo, Jianfeng Qiu

**Affiliations:** 1 College of Bee Science and Biomedicine, Fujian Agriculture and Forestry University, Fuzhou 350002, China; zhangyiqiong1121@163.com (Y.Z.); 13277017210@163.com (M.W.); xwenhua0911@163.com (W.X.); zanghe321@163.com (H.Z.); 18133433927@163.com (T.W.); 19988583429@163.com (K.H.); dfchen826@fafu.edu.cn (D.C.); 2 National & Local United Engineering Laboratory of Natural Biotoxin, Fuzhou 350002, China; yantizhen@gdmu.edu.cn (T.Y.); luoqingxian@163.com (Q.L.); 3 Apitherapy Research Institute of Fujian Province, Fuzhou 350002, China

**Keywords:** honeybee, *Apis cerana*, *Nosema ceranae*, piRNA, target, immune response

## Abstract

Piwi-interacting RNAs (piRNAs) play an essential part in transposon suppression, DNA methylation, and antiviral responses. The current understanding of the roles of piRNAs in honeybees is very limited. This study aims to analyze the expression pattern and regulatory role of piRNAs in the Asian honeybee (*Apis cerana*) responding to infection by *Nosema ceranae*, based on previously gained small RNA-seq data. Here, 450 and 422 piRNAs were respectively identified in the midgut tissues of *Apis cerana cerana* workers at 7 and 10 days post-inoculation (dpi) with *N. ceranae*, including 539 non-redundant ones. Additionally, one up-regulated (piR-ace-1216942) and one down-regulated (piR-ace-776728) piRNA were detected in the workers’ midgut at 7 dpi, targeting 381 mRNAs involved in 31 GO terms, such as metabolic processes, catalytic activity, and organelles, as well as 178 KEGG pathways, including lysosome, MAPK signaling pathway, and purine metabolism. A total of 35 up-regulated and 11 down-regulated piRNAs were screened from the workers’ midgut at 10 dpi, targeting 13,511 mRNAs engaged in 50 GO terms, such as biological regulation, transporter activity, and membrane, as well as 389 KEGG pathways, including the JAK-STAT signaling pathway, Hippo signaling pathway, and nitrogen metabolism. Further analysis indicated that 28 differentially expressed piRNAs (DEpiRNAs) in the midgut at 10 dpi could target 299 mRNAs annotated to three cellular immune pathways (lysosome, endocytosis, and phagosome), while 24 DEpiRNAs could target 205 mRNAs relevant to four humoral immune pathways (FoxO, JAK-STAT, NF-*κ*B, and MAPK signaling pathway). Through Sanger sequencing and RT-qPCR, the expression of six randomly selected DEpiRNAs was verified. Moreover, the dual-luciferase reporter gene assay confirmed the binding relationships between piR-ace-446232 and *CRT* as well as between piR-ace-1008436 and *EGFR*. Our findings not only contribute to enrich our understanding of the role of piRNAs in honeybees but also provide a basis for exploring the host response to *N. ceranae* infection mediated by piRNAs.

## 1. Introduction

Non-coding RNAs (ncRNAs), including small ncRNAs like Piwi-interacting RNA (piRNA) and microRNA (miRNA) as well as long non-coding RNA (lncRNA) and circular RNA (circRNA), are capable of modulating a wide range of essential processes, such as embryonic development, tissue differentiation, and organogenesis [1]. Among various types of ncRNAs, piRNAs are a class of small ncRNAs abundantly expressed in germ cells, with a length distribution of 24–34 nt [2]. PiRNAs were first identified in mouse germ cells [3] and later detected in a variety of model organisms, including insects such as *Drosophila melanogaster* [4], *Aedes albopictus* [5], and *Bombyx mori* [6]. piRNAs were initially recognized as key regulators of transposon silencing [7] and maintenance of genomic integrity [8]. A growing body of evidence has suggested that piRNAs are involved in the regulation of an array of biological processes, including mRNA stability, protein synthesis, chromatin organization, DNA methylation [9], epigenetic regulation [10], genome structure, growth and development [11], and antiviral responses [12], primarily by binding to Argonaute family proteins, among other mechanisms [13].

*Apis cerana cerana* is the nominate subspecies of *A. cerana* and one of the most prevalent bee species used in beekeeping in many Asian countries including China. Microsporidia are a kind of unicellular fungi that reproduce intracellularly and have a broad host range, infecting both vertebrates and invertebrates [14]. *Nosema ceranae* is a microsporidian parasite that infects the epithelial cells of the midgut of adult bees and is one of the primary pathogens responsible for microsporidiosis in honeybees [15]. *N. ceranae* infection not only causes shortened lifespan, energy stress, and immunosuppression in bee hosts but also harms bee colonies in combination with other biotic or abiotic stress, giving rise to severe losses in the apicultural industry [16,17].

As compared with human and model organisms like mouse and fruitfly, piRNAs in the majority of insects, including honeybees, are largely unknown. Previous studies have suggested the relevance between piRNAs and a subseries of biological processes of *A. mellifera*, such as sex determination [18], gene regulation [19], silence transposons [20], gut development [21], and response to fungal invasion [22]. For instance, Wang et al. [20] documented that the elevated expression of piRNA in drones might serve to repress transposons, thereby mitigating the impact of transposon-related genetic instability. Watson et al. [18] reported that elevated piRNA levels in semen relative to the *A. mellifera* eggs and ovaries coupled with the identification of a considerable number of piRNAs that target sex-determining motifs point towards a potential involvement of piRNAs in the process of sex determination in honeybees.

Recently, the extensive negative influence of *N. ceranae* infection on *A. cerana* workers was reported [23], and the host response to fungal invasion mediated by miRNAs [24], lncRNAs [25], and circRNAs [26] was deciphered. Here, to explore the regulatory role of piRNAs in *A. c. cerana* workers in response to *N. ceranae* infection, piRNAs in the midgut tissues of *A. c. cerana* workers inoculated with *N. ceranae* were identified, based on sRNA-seq and bioinformatics. Additionally, differentially expressed piRNAs (DEpiRNAs) were screened and analyzed to investigate their expression profile, regulatory network, and putative function. Moreover, the molecular validation of DEpiRNAs was conducted, followed by verification of the binding relationships between two DEpiRNAs and corresponding target mRNAs. The findings from this work not only offer candidate molecules for further functional dissection but also illustrate the potential DEpiRNA-modulated mechanism underlying the response of *A. c. cerana* workers to *N. ceranae* invasion.

## 2. Results

### 2.1. Quantity, Property, and Expression Pattern of piRNAs in the Midguts of A. c. cerana Workers Following N. ceranae Invasion

Following *N. ceranae* challenge, 450 and 422 piRNAs were identified in the midgut tissues of *A. c. cerana* workers at 7 dpi and 10 dpi, respectively. After removing redundant ones, 539 distinct piRNAs in total were identified. Among these, 333 piRNAs were shared by both groups, while the quantities of unique ones were 117 and 89, respectively. Further analysis revealed that the top three most highly expressed piRNAs in the workers’ midgut at 7 dpi were piR-ace-992993 (TPM = 15,274.08), piR-ace-326296 (TPM = 14,750.54), and piR-ace-903081 (TPM = 14,512.62). In the workers’ midgut at 10 dpi, the most highly expressed piRNA was piR-ace-903081 (TPM = 15,716.15), followed by piR-ace-263349 (TPM = 15,710.26) and piR-ace-119875 (TPM = 15,695.53).

**Figure 1:**
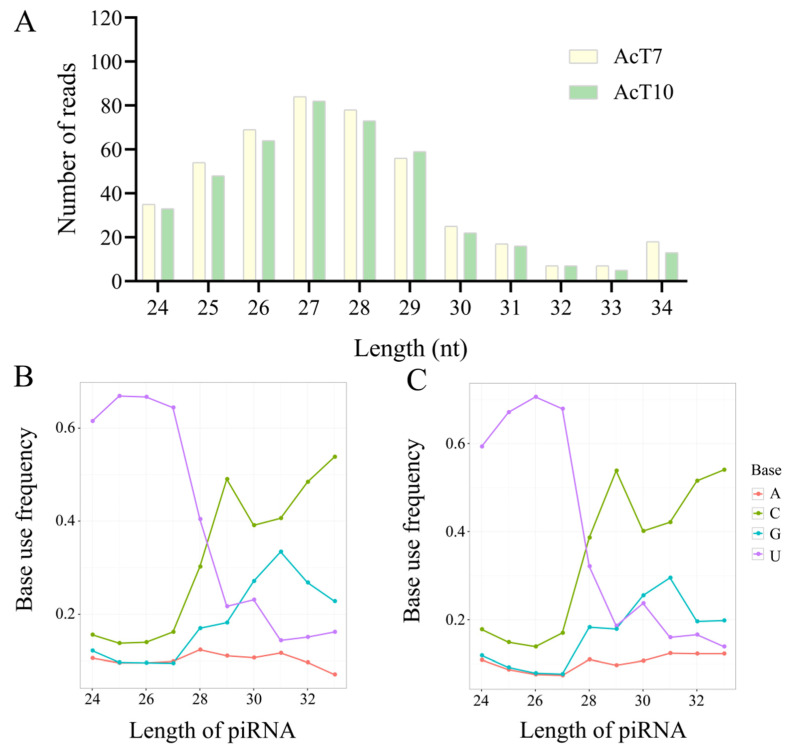
Length distribution and first base bias of piRNAs discovered in *N. ceranae*-inoculated *A. c. cerana* workers. (**A**) Length distribution of piRNAs expressed in the Ac7T and Ac10T groups. (**B**) First base bias of piRNAs identified in the Ac7T group. (**C**) First base bias of piRNAs identified in the Ac10T group.

The length distribution of piRNAs in the Ac7T and Ac10T groups ranged from 24 nt to 34 nt, with the most abundant length being 27 nt, followed by 28 nt and 26 nt (Figure 1A). In the Ac7T group, those piRNAs with a length distribution of 24–28 nt exhibited a strong bias toward uracil (U) at the first base, while those distributed between 29 nt and 33 nt in length had a cytosine (C) bias at the first base (Figure 1B). A similar phenomenon was observed for piRNAs identified in the Ac10T group, as shown in Figure 1C.

### 2.2. Expression Profile of piRNAs in A. c. cerana Worker Midguts Following N. ceranae Inoculation

**Figure 2:**
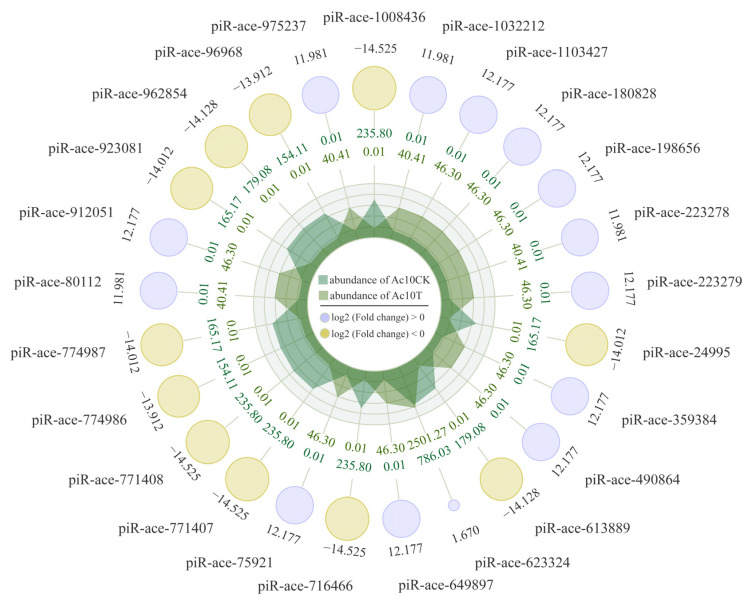
Radar plot of the top 25 DEpiRNAs in the Ac10CK vs. Ac10T comparison. Yellow circles represent down-regulated piRNAs, while purple circles represent up-regulated piRNAs. The size of each circle corresponds to the magnitude of the log_2_FC, with larger circles indicating a greater difference. Data outside the circles reflect the average expression levels of piRNAs in the Ac10CK group, while data within the circles represent the average expression in the Ac10T group. Light and dark green regions at the center indicate the relative expression abundance of piRNAs in Ac10CK and Ac10T, respectively, along each axis.

In the Ac7CK vs. Ac7T comparison, one up-regulated piRNA (piR-ace-1216942, log_2_FC = 12.58, *p* = 1.00 × 10^−5^) and one down-regulated one (piR-ace-776728, log_2_FC = −11.24, *p* = 2.66 × 10^−6^) were detected. In contrast, there were 35 up-regulated and 11 down-regulated piRNAs identified in the Ac10CK vs. Ac10T comparison group; the top three up-regulated piRNAs were piR-ace-1032212 (log_2_FC = 11.98, *p* = 4.73 × 10^−7^), piR-ace-223278 (log_2_FC = 11.98, *p* = 4.73 × 10^−7^), and piR-ace-80112 (log_2_FC = 11.98, *p* = 4.73 × 10^−7^), whereas the three most down-regulated piRNAs were piR-ace-774986 (log_2_FC = −13.91, *p* = 5.40 × 10^−4^), piR-ace-96968 (log_2_FC = −13.91, *p* = 5.40 × 10^−4^), and piR-ace-613889 (log_2_FC = −14.13, *p* = 5.43 × 10^−4^) (Figure 2).

### 2.3. Annotation of mRNAs Targeted by DEpiRNAs

**Figure 3:**
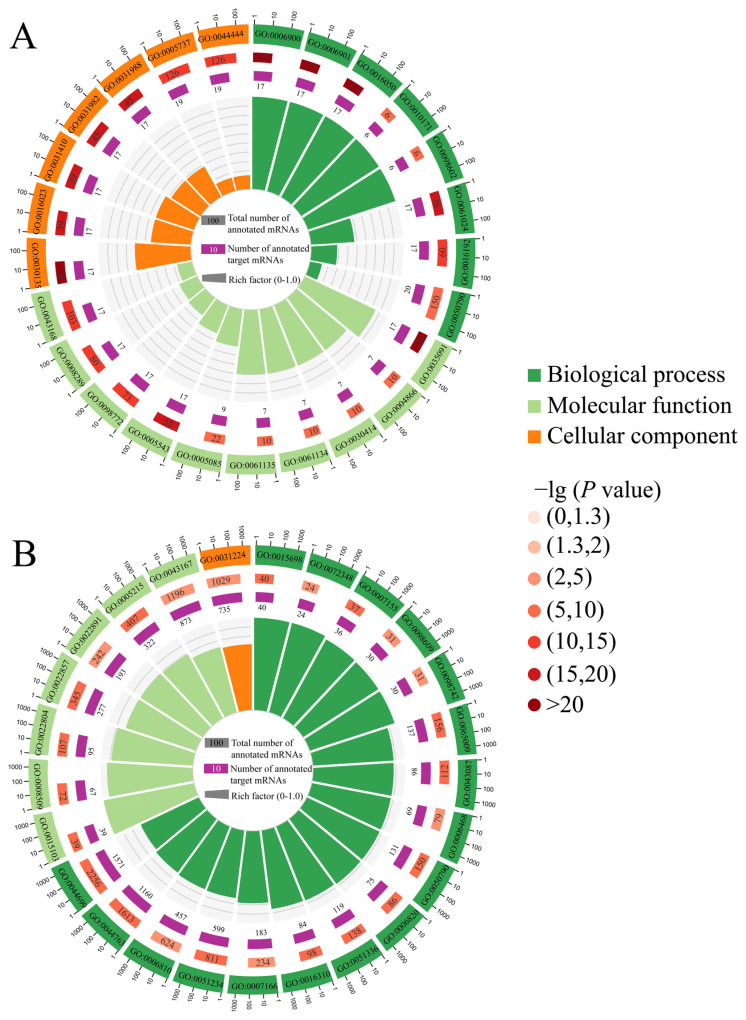
Loop graphs of target mRNAs targeted by DEpiRNAs in the *A. c. cerana* workers’ midguts at 7 dpi (**A**) and 10 dpi (**B**) with *N. ceranae*. From outside to inside, the first circle represents the enriched terms with GO numbers in the rectangles, with different colors corresponding to different GO categories, dark green for biological process, light green for molecular function and orange for cellular component; the next circles indicate the number of target mRNAs enriched in different GO categories in contrast to the background genes and corresponding *p*-values—the darker the orange color, the larger the number of target mRNAs and the smaller the *p*-values; the innermost circle represents the set criteria for the quantity of target mRNAs, with different colors representing different GO categories; rich factor means the number of foreground target mRNAs enriched in GO categories divided by the number of background genes.

In the Ac7CK vs. Ac7T comparison, one DEpiRNA (piR-ace-776728) was predicted to target 381 mRNAs annotated to 31 Gene Ontology (GO) terms, including fourteen biological process-related terms such as cellular processes and metabolic processes, seven molecular function-relative terms such as catalytic activity and binding, and ten cellular component-relevant terms like cell and organelle (Figure 3A). Comparatively, 43 DEpiRNAs in the Ac10CK vs. Ac10T comparison could target 13,511 mRNAs involved in 23 functional terms related to biological processes including biological regulation and single-organism processes, 10 molecular function-associated terms such as transporter activity and molecular function regulation, and 17 cellular component-relevant terms like membrane and extracellular matrix (Figure 3B).

**Figure 4:**
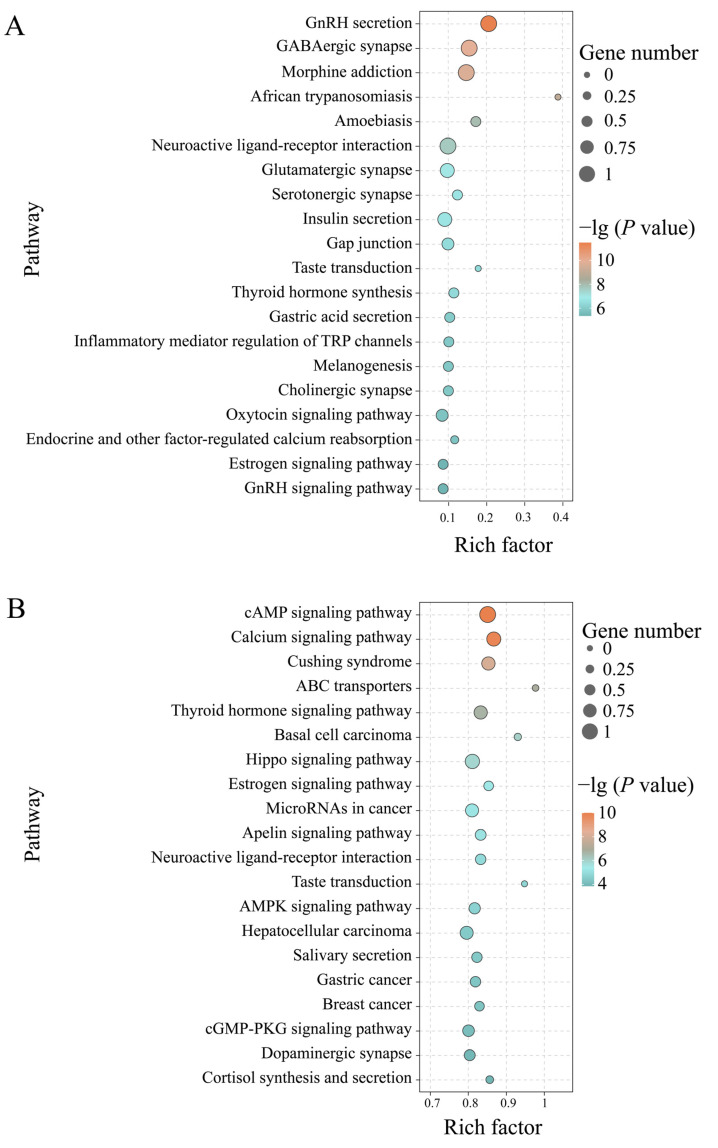
Bubble diagrams (Top 20) of DEpiRNA-targeted mRNAs in the *A. c. cerana* workers’ midguts at 7 dpi (**A**) and 10 dpi (**B**) with *N. ceranae*.

In addition, the target mRNAs in the Ac7CK vs. Ac7T comparison group were found to be engaged in 178 Kyoto Encyclopedia of Genes and Genomes (KEGG) pathways relative to cellular processes, environmental information processing, genetic information processing, human diseases, metabolism, and organismal systems. Among these, some pathways, like lysosome, GnRH signaling pathway, endocytosis, MAPK signaling pathway, and purine metabolism, were enriched by target mRNAs (Figure 4A). In contrast, DEpiRNAs in the Ac10CK vs. Ac10T comparison group were associated with 389 pathways, such as the cAMP signaling pathway, fructose and mannose metabolism, Hippo signaling pathway, JAK-STAT signaling pathway, and nitrogen metabolism (Figure 4B).

### 2.4. Regulatory Networks Between DEpiRNAs and Target mRNAs Regarding Cellular and Humoral Immune Pathways

**Figure 5:**
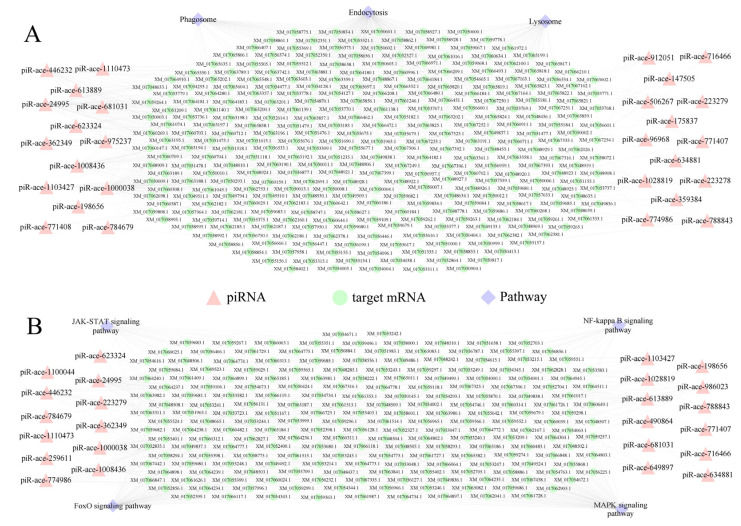
Cellular (**A**) and humoral (**B**) immune pathway-associated regulatory networks between DEpiRNAs and target mRNAs in the midgut of *A. c. cerana* workers at 10 dpi with *N. ceranae.* Pink triangles represent piRNAs, green circles represent target mRNAs, and purple diamonds represent pathways.

Regulatory network analysis showed that 28 DEpiRNAs in the Ac10CK vs. Ac10T comparison group could target 299 mRNAs involved in three cellular immune pathways, including lysosome, endocytosis, and phagosome (Figure 5A), while 24 DEpiRNAs could target 205 mRNAs associated with four humoral immune pathways, such as FoxO, JAK-STAT, NF-*κ*B, and MAPK signaling pathways (Figure 5B).

### 2.5. Molecular Validation and RT-qPCR Detection of DEpiRNAs

**Figure 6:**
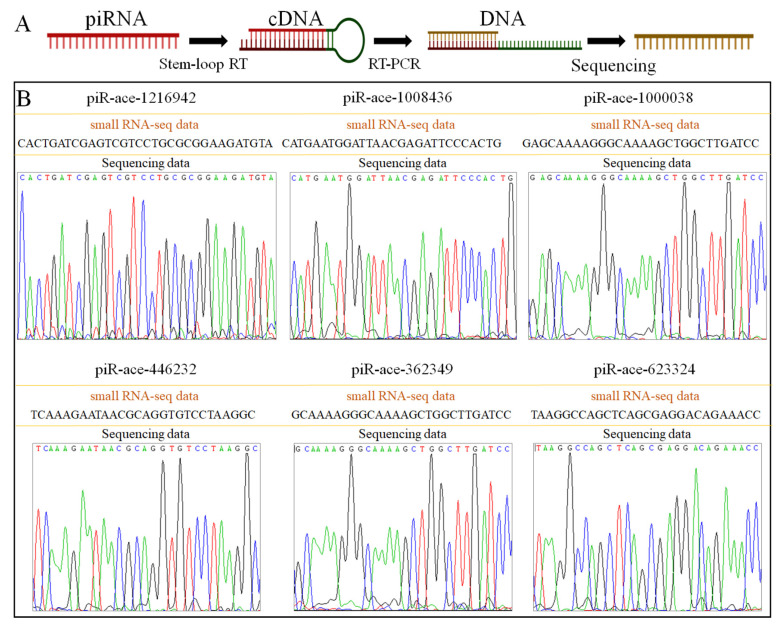
The schematic map (**A**) and peak diagram of Sanger sequencing (**B**) of the amplified fragments from six *A. c. cerana* DEpiRNAs.

Six DEpiRNAs (piR-ace-1216942, piR-ace-1008436, piR-ace-1000038, piR-ace-446232, piR-ace-362349, and piR-ace-623324) were amplified by stem-loop RT-PCR and Sanger sequencing (Figure 6A). The results showed that the nucleotide sequences of the DEpiRNAs were consistent with the small RNA-seq data, confirming that the sequences of the piRNAs were correct (Figure 6B).

**Figure 7:**
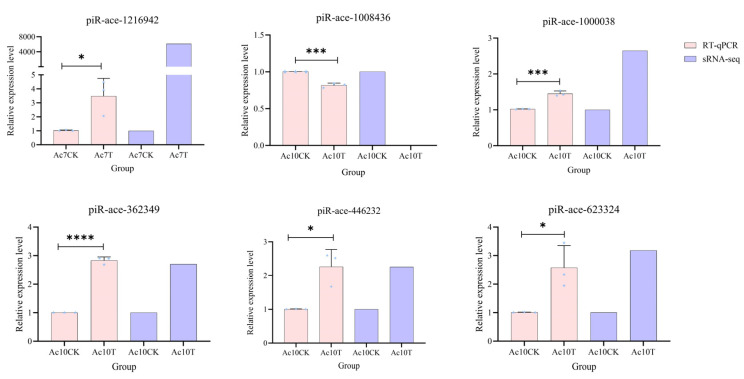
RT-qPCR detection of six DEpiRNAs. *, *p* < 0.05; ***, *p* < 0.001; ****, *p* < 0.0001. piR-ace-1216942 was a DEpiRNA in the Ac7CK vs. Ac7T comparison group, whereas the other five DEpiRNAs were selected from the Ac10CK vs. Ac10T comparison group. The blue pentagrams represent the expressions corresponding to each biological repetition.

The expression patterns of these six DEpiRNAs were further verified by RT-qPCR after microsporidium inoculation. The results showed that the expression of piR-ace-1216942 was significantly up-regulated at 7 dpi. The expressions of piR-ace-1000038, piR-ace-362349, piR-ace-446232, and piR-ace-623324 were significantly up-regulated at 10 dpi. The expression of piR-ace-1008436 was significantly down-regulated at 10 dpi. The expression patterns of these DEpiRNAs were consistent with the data from small RNA-seq, verifying the reliability of the transcriptome datasets used in this study (Figure 7).

### 2.6. Confirmation of the Binding Relationships Between piR-ace-446232 and CRT as Well as Between piR-ace-1008436 and EGFR

**Figure 8:**
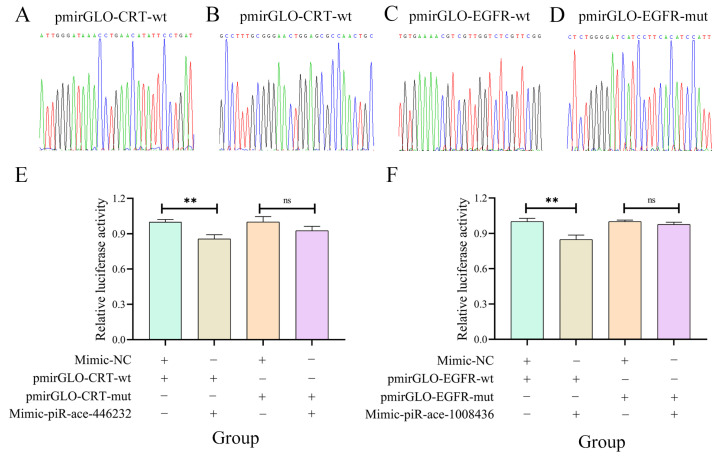
Verification of binding relationships between piR-ace-446232 and *CRT* and between piR-ace-1008436 and *EGFR* by dual-luciferase reporter gene assay. (**A**,**B**): Peak diagrams of Sanger sequencing of binding site between piR-ace-446232 and *CRT* and corresponding mutation site. (**E**): Dual-luciferase reporter gene assay of the binding relationship between piR-ace-446232 and *CRT*, Columns of different colours represent relative luciferase activity under different treatments, the same below. (**C**,**D**): Peak diagrams of Sanger sequencing of binding site between piR-ace-1008436 and *EGFR* and corresponding mutation site. (**F**): Dual-luciferase reporter gene assay of the binding relationship between piR-ace-1008436 and *EGFR*. Data in the figure are mean ± SE. Symbols above bars indicate significant difference between two groups (ns, *p* > 0.05; **: *p* < 0.01, Student’s *t* test).

The results of Sanger sequencing were indicative of the successful construction of the recombinant plasmids pmirGLO-CRT-wt, pmirGLO-CRT-mut, pmirGLO-EGFR-wt, and pmirGLO-EGFR-mut (Figure 8A–D). Dual-luciferase assays revealed a significant reduction in cell fluorescence activity (*p* < 0.01) in the mimic-piR-ace-446232 and pmirGLO-CRT-wt co-transfected group as compared to the mimic-NC and pmirGLO-CRT-wt co-transfected group. Additionally, a non-significant change (*p* > 0.05) in fluorescence activity was observed between the mimic-piR-ace-446232 and pmirGLO-CRT-mut co-transfected group as well as between the mimic-NC and pmirGLO-CRT-mut co-transfected group. Similarly, the fluorescence activity in the mimic-piR-ace-1008436 and pmirGLO-EGFR-wt co-transfected group was significantly decreased (*p* < 0.01) in comparison with the mimic-NC and pmirGLO-EGFR-wt co-transfected group (Figure 8E), while there was a non-significant difference (*p* > 0.05) between the mimic-piR-ace-1008436 and pmirGLO-EGFR-mut co-transfected group and between the mimic-NC and pmirGLO-EGFR-mut co-transfected group (Figure 8F). These results confirmed the binding interactions between piR-ace-446232 and *CRT* as well as between piR-ace-1008436 and *EGFR.*

## 3. Discussion

### 3.1. Identification of piRNA in Apis cerana cerana After Infestation by Nosema ceranae Microsporidia and Dynamic Changes in Expression Patterns

In this current work, 539 piRNAs were identified in the midguts of *A. c. cerana* workers at 7 dpi and 10 dpi with *N. ceranae*, with 333 shared ones. Considering the limited information about *A. cerana* piRNAs, the identified piRNAs, together with 602 piRNAs previously discovered in the un-inoculated midguts, offered a comprehensive catalog of *A. c. cerana* piRNAs, providing a valuable resource for continuous and deep investigation of their functions and mechanisms. In addition, structural characterization revealed that the piRNAs in both the Ac7T and Ac10T groups were predominantly distributed ranging from 24 nt to 29 nt in length, which closely resembled the length distribution of piRNAs from other species, including *Drosophila melanogaster* [27], *Danio rerio* [28], *Mus musculus* [29], and *Oryza sativa* [30]. This suggests that piRNAs across diverse plant and animal species share a high degree of structural conservation.

In the workers’ midgut at 7 dpi, one up-regulated and one down-regulated piRNA were identified, while 35 up-regulated and 11 down-regulated ones were detected in the workers’ midgut at 10 dpi, respectively. This indicates that *N. ceranae* infestation triggers differential piRNA expression in the midgut tissues of *A. c. cerana* worker bees; as the infestation was prolonged, more piRNAs were observed to regulate the host response, with the intensity of the response also increasing.

### 3.2. DEpiRNA May Be Involved in Regulating the Immune Response of Apis cerana cerana to Nosema ceranae by Targeting mRNAs

piRNAs can bind to the mRNAs of specific target genes, thereby affecting their translation as well as stability, ultimately regulating gene expression [31]. Here, the target mRNAs of DEpiRNAs were found to be enriched in an array of functional terms, like biological regulation, positive regulation of biological processes, metabolic processes, and catalytic activity. Also, these targets were associated with a series of vital pathways, including amino sugar and nucleotide sugar metabolism, circadian rhythm, metabolic pathways, and dorso-ventral axis formation. Together, these results indicate that DEpiRNAs may mediate host cellular activities and biological processes of importance during the *N. ceranae* infestation through regulation of the above-mentioned critical terms and pathways.

Biological adhesion has been proposed as an indirect regulator of the immune response by influencing immune cell adhesion, migration, and interactions [32]. Cell killing protects the body via the elimination of pathogens, cancerous cells, and other abnormal cells, thereby preventing further infection [33]. Antioxidant activity refers to the ability to inhibit oxidative reactions in molecules or substances. Antioxidants protect immune cells against free radical damage, ensuring their optimal function [34]. Hence, maintaining adequate antioxidant intake is crucial for a healthy immune system [34]. In the present study, seven mRNAs in the midgut at 7 dpi were enriched in the biological adhesion category, whereas fourty-nine, five, four, and one mRNA were respectively enriched in the categories of biological adhesion, antioxidant activity, immune system processes, and cell killing. These findings suggest that, as *N. ceranae* proliferation progressed, the stress on the midgut intensified during the later stages of infestation, suggesting that the workers’ immune defenses continued to improve.

In response to infections by pathogenic micro-organisms, insects have developed an efficient natural immune system over a long period of evolution, and the immune defense of honeybees can be divided into a colony-level and individual-level immune response [35], the latter being subdivided into a cellular and humoral immune response [36]. The insect cellular immune system primarily consists of hemolymph-mediated processes, such as phagocytosis, nodulation, encapsulation, and melanisation [36]. Here, five target mRNAs in the midgut at 7 dpi with *N. ceranae* were involved in lysosome and leukocyte transendothelial migration, two target mRNAs were engaged in endocytosis, and one mRNA was enriched in phagosome. At 10 dpi with *N. ceranae*, the number of host mRNAs relative to cellular immune pathways was increased, with 187, 101, 81, 74, and 55 mRNAs enriched in endocytosis, ubiquitin-mediated proteolysis, adherens junctions, lysosome, and phagosome, respectively. These results demonstrate that the midgut of *A. c. cerana* worker bees exhibited sustained activation and enhancement of cellular immunity in response to prolonged *N. ceranae* stress. The Toll, IMD, and JAK/STAT signaling pathways are vital humoral immune pathways in insects [36]. A significant number of bacteria and viruses are able to trigger an immune response through the NF-*κ*B signaling pathway, which regulates the expression of numerous inflammatory cytokines, chemokines, immune receptors, and activated cell surface adhesion molecules [37]. The FoxO signaling pathway may play a part in the regulation of apoptosis [38]. The JAK/STAT signaling pathway is a highly conserved pathway across diverse insects and serves as the primary immune mechanism for resisting pathogen invasion. Additionally, it is a pivotal component of the Toll and IMD signaling pathways. Its role in insect immunity, hormone regulation, and other physiological processes is of considerable significance [39,40]. PIWIs/piRNAs through PI3K/Akt signaling pathway, STAT signaling pathway, TGF-β signaling pathway, and Fas signaling pathway for pro-apoptotic or anti-apoptotic effects in cells [41]. In previous studies, piR-ame-1128833 was discovered to be a potential regulator of the MAPK signaling pathway and the JAK-STAT pathway, which may contribute to the immune response to *N. ceranae* in *A. mellifera* [22]. Here, it is observed that seven and five mRNAs in the Ac7CK vs. Ac7T comparison group were respectively involved in the PI3K-Akt and MAPK signaling pathways, whereas in the Ac10CK vs. Ac10T comparison group, 134, 120, 30, and 20 mRNAs were engaged in the PI3K-Akt, MAPK, JAK-STAT, and NF-*κ*B signaling pathways, respectively. The results suggest that the corresponding DEpiRNAs may influence the response of *A. c. cerana* workers to *N. ceranae* infection by modulating the humoral immune pathways mentioned above. A similarity has been observed between the signaling pathways of insects and mammals, including those associated with cancer. Studies have shown that up to 75% of disease-related genes in humans have functional *Drosophila* orthologs [42,43]. When cancer-related genes of *Drosophila* are perturbed, the flies exhibit classic hallmarks of cancer, such as escape from apoptosis, sustained proliferation, and metabolic reprogramming [44,45]. The current model of *Drosophila* cancer has been utilized to address fundamental aspects of human cancer, such as clonal evolution, the tumor’s microenvironment, cancer cachexia, and resistance to anticancer drugs [46]. In the Ac10CK and Ac10T comparison groups, 137, 100, and 88 mRNAs were found to be involved in hepatocellular carcinoma, gastric cancer, and breast cancer signaling pathways, respectively. These signaling pathways may be immune responses or other physiological characteristics associated with cancer.

In summary, these findings indicate that the immune system of *A. c. cerana* workers recognizes pathogen invasion and responds with both cellular and humoral immunity during the early stages of *N. ceranae* invasion and becomes further activated as fungal infection progresses.

### 3.3. piR-ace-446232 and piR-ace-1008436 May Be Modulators of the Host Immune Response

This study successfully confirmed the binding relationships between piR-ace-446232 and *CRT* and between piR-ace-1008436 and *EGFR* using a dual-luciferase assay. Calreticulin has been shown to serve a crucial function in the phagocytosis of insects [47]. Wang et al. [48] identified a *CRT* in the venom of *Pteromalus puparum*, which protected the offspring of the host against host cellular immune responses; this is achieved via the functional component PpCRT, which inhibits the expression of host cellular response-related genes. Here, piR-ace-446232 was verified to target *CRT*, thereby modulating cellular immune pathways, which may influence the response of worker bees to *N. ceranae* invasion. The epidermal growth factor receptor plays a critical role in maintaining epithelial cell homeostasis [49]. Jin et al. [50] reported that the activation of the ERK and PI3K/Akt signaling pathways in the silkworm *B. mori*, through the regulation of the *B. mori* epidermal growth factor receptor (BmEGFR), triggers an immune response to baculovirus. It is hypothesized that piR-ace-1008436 was likely to be a key regulator in the host response to *N. ceranae* infection by targeting *EGFR*. The regulatory functions and potential mechanisms of the piR-ace-446232–*CRT* and piR-ace-1008366–*EGFR* axes will continue to be investigated in the future to further identify new targets for controlling nosemosis in the apicultural industry.

## 4. Materials and Methods

### 4.1. Biological Materials

Fresh spores were isolated from a heavily infected *A. cerana* colony in an apiary located in Fuzhou, Fujian Province, China, using the method described in the published study [51,52]. Three *Nosema*-free (confirmed by PCR) bee colonies were selected from the experimental apiary of the College of Bee Science and Biomedicine of Fujian Agriculture and Forestry University. Three foundation frames of sealed brood combs from each colony were quickly transferred to incubators and cultured at 34 ± 2 °C for 24 h to provide newly emerged workers. We divided the newly emerged bees into 3 groups, inoculating 20 workers in each group, for a total of 60 workers. A total of 5 μL of a 50% sucrose solution containing 1 × 10^6^ fresh spores of *N. ceranae* was individually fed into the proboscis of workers until the solution was completely ingested, while the control groups (CK) were fed with 5 μL of the same sucrose solution without spores. At 2 h after inoculation, a spore-free sucrose solution was randomly fed, and the feeders were replaced daily. The midguts of the *N. ceranae*-treated and control workers were harvested at 7 days post-inoculation (7 dpi) and 10 dpi, respectively, and then immediately frozen in liquid nitrogen. The 7 dpi corresponds to the proliferation phase of the spores, and 10 dpi marks the maturation and release stage of the spores. Three midguts were pooled into a group, with each group consisting of three biological replicates. The *N. ceranae*-treated groups at 7 dpi and 10 dpi were labeled as Ac7T and Ac10T, while the control groups at 7 dpi and 10 dpi were labeled as Ac7CK and Ac10CK, respectively.

### 4.2. Source of sRNA-Seq Data

The midgut tissues of *N. ceranae*-inoculated *A. c. cerana* workers at 7 dpi and 10 dpi along with corresponding un-inoculated workers were prepared following the established protocol developed by [53], followed by RNA extraction, cDNA library construction, deep sequencing using the Illumina MiSeq platform (Genedenovo Biotechnology Co., Ltd., Guangzhou, China), and strict quality control of the raw data [53]. The quality control results demonstrated that a total of 127,523,419 raw reads were generated, of which 122,104,443 were clean reads, representing 95.75% of the total number of reads. Raw data are available in the NCBI Short Read Archive (http://www.ncbi.nlm.nih.gov/sra/ (accessed on 16 December 2024)) under BioProject number PRJNA487111 [52].

### 4.3. Bioinformatic Prediction and Analysis of piRNAs

*A. c. cerana* piRNAs were identified according to the previously described protocol [21]. Briefly, (1) the clean reads were mapped to the *Apis creana* genome (GCF_001442555.1_ACSNU-2.0) to obtain mapped reads; (2) the mapped clean tags were aligned to the GenBank [54] and Rfam [55] databases using the blastn (version 2.2.25) tool to remove rRNA, scRNA, snoRNA, snRNA, and tRNA; (3) the remaining clean reads were compared to the miRBase database [56] using bowtie2 software (version 2.5.4) (Johns Hopkins University, Baltimore, MD, USA) [57], and new miRNAs were identified using MiRdeep2 software (version 2.0.1.2) [58] and removed; (4) sRNAs with a length distribution between 24 nt and 33 nt were screened on the basis of the length characteristics of piRNAs, and only those aligned to a unique position were retained as candidate piRNAs; (5) candidate piRNAs were further compared to piRBase using bowtie2 software (version 2.5.4) (Johns Hopkins University, Baltimore, MD, USA) [59] to obtain piRNAs present in the database. Next, the length distribution bowtie and first base bias of the piRNAs were investigated based on the prediction result [60].

### 4.4. Identification of DEpiRNAs and the Prediction of Its Target mRNAs

The expression level of each piRNA was normalized to tags per million (TPM) using the formula TPM = (T × 10^6^)/N, where T represents the number of piRNA clean reads and N represents the total number of sRNA clean reads [5]. The fold change in the expression level of each piRNA between different groups was calculated using the formulas (TPM in Ac7CK)/(TPM in Ac7T) and (TPM in Ac10CK)/(TPM in Ac10T). DEpiRNAs in the Ac7CK vs. Ac7T and Ac10CK vs. Ac10T comparison groups were screened using edgeR software (http://www.bioconductor.org/ (version 4.2 accessed on 16 December 2024)), based on the criteria of |log_2_ fold change (log_2_FC)| ≥ 1 and *p* ≤ 0.05 [61].

TargetFinder software (https://github.com/carringtonlab/TargetFinder (accessed on 16 December 2024)) was used to predict the target genes of the DEpiRNAs [62], with default parameters applied. The predicted targets were aligned to the GO (https://www.geneontology.org/ (accessed on 16 December 2024)) and KEGG (https://www.genome.jp/kegg/ (accessed on 16 December 2024)) databases using the BLAST tool (https://blast.ncbi.nlm.nih.gov/Blast.cgi (accessed on 16 December 2024)) to obtain the corresponding annotations. Based on the predicted targeting relationships, regulatory networks between DEpiRNAs and their target mRNAs were constructed, with a threshold of free energy < −10 kJ/mol, and visualized using Cytoscape 3.8.2 software [63].

### 4.5. Stem-Loop RT-PCR Amplification and Sanger Sequencing of the DEpiRNAs

One piRNA (piR-ace-1216942) from the Ac7CK vs. Ac7T comparison group and five piRNAs (piR-ace-1000038, piR-ace-623324, piR-ace-1008436, piR-ace-446232, and piR-ace-362349) from the Ac10CK vs. Ac10T comparison group were randomly selected for RT-PCR validation. Total RNA was isolated from the midgut tissues of 7- and 10-dpi workers using the SteadyPure Quick RNA Extraction Kit (Accurate, Changsha, China), and its purity and concentration were assessed using a Nanodrop 2000 spectrophotometer (Thermo Fisher, Waltham, MA, USA). Specific stem-loop primers along with upstream primers (F) and universal downstream primers (R) (Table S1) were designed using DNAMAN software (Version: 8.0.8.789) and synthesized by Sangon Biotech Co., Ltd. (Shanghai, China). Reverse transcription was performed using the HiScript^®^ 1st Strand cDNA Synthesis Kit (Yeasen, Shanghai, China) according to the manufacturer’s instructions, and the resulting cDNAs were used as templates for PCR amplification of DEpiRNAs on a T100 thermocycler (Bio-Rad, Hercules, CA, USA). The PCR system and procedure were set according to the method described by Sun et al. [60]. The amplification products were analyzed by 1.8% agarose gel electrophoresis using GoldView staining (Accurate, Beijing, China) and detected with a Bio-Rad ChemiDoc XRS system (Peiqing, Shanghai, China). The target fragments were identified by electrophoresis, isolated, and subjected to TA cloning. A small amount of bacterial culture was used for PCR identification, and positive clones were sent to Sangon Biotech (Shanghai) Co., Ltd. (Sangon, Shanghai, China) for Sanger sequencing.

### 4.6. RT-qPCR Verification of DEpiRNAs

The RT-qPCR assays of these six DEpiRNAs mentioned above were carried out according to the protocol of the SYBR Green Dye kit (Yeasen, Shanghai, China). The cDNA prepared in Section 4.6 was used as templates for the DEpiRNAs. Reverse transcription was carried out using a mixture of random primers and oligo(dT) primers, and the resulting cDNA was subsequently used as templates for qPCR of the reference gene snRNA *U*6 (GenBank accession number: LOC107992425). The reaction system and procedure were set following the documentation by Sun et al. [64]. There were three replicates for each reaction. The relative expression level of each DEpiRNA was calculated with the 2^−ΔΔCt^ method [65]. Student’s *t*-test of the qPCR data was conducted with Graph Prism 8 software [66].

### 4.7. Dual-Luciferase Assay

According to the method described by Fan et al. [67], the mimic-piRs of piR-ace-446232 and piR-ace-1008436 (mimic-piR-ace-446232 and mimic-piR-ace-1008436) and the corresponding negative control mimic (mimic-NC) were designed and synthesized by Shanghai Gemma Pharmaceutical Technology Co (Gemma, Shanghai, China). The potential binding sites of piR-ace-446232 with *calreticulin* (*CRT*) (GenBank accession number: LOC108003097) and piR-ace-1008436 with *epidermal growth factor receptor* (*EGFR*) (GenBank accession number: LOC107993429) were predicted using RNAhybrid software (https://bibiserv.cebitec.uni-bielefeld.de/rnahybrid (accessed on 16 December 2024)) [68]. Specific upstream and downstream primers were designed for PCR amplification based on the nucleotide sequences of the binding sites, and the amplified fragments were cloned into pmirGLO vectors, resulting in pmirGLO-CRT-wt and pmirGLO-EGFR-wt, respectively. The mutant sequences of the binding sites were also designed, synthesized, and cloned into pmirGLO vectors, resulting in pmirGLO-CRT-mut and pmirGLO-EGFR-mut, respectively. The bacterial cultures were sent to Sangon Biotech (Shanghai) Co., Ltd. (Sangon, Shanghai, China) for Sanger sequencing, and correctly sequenced plasmids were transferred to fresh LB liquid medium. Plasmids were extracted using the Endotoxin Removal Plasmid Extraction Kit (Total Gold, Beijing, China). HEK-293T cells were seeded in 96-well plates and cultured at 37 °C for 24 h to achieve 90–95% confluency. Transfection was performed according to the instructions of the Hieff Trans™ Liposomal Nucleic Acid Transfection Reagent (Yeasen, Shanghai, China), with eight co-transfection groups: (a) mimic-piR-ace-446232 and pmirGLO-CRT-wt; (b) mimic-NC and pmirGLO-CRT-wt; (c) mimic-piR-ace-446232 and pmirGLO-CRT-mut; (d) mimic-NC and pmirGLO-CRT-mut; (e) mimic-piR-ace-1008436 and pmirGLO-EGFR-wt; (f) mimic-NC and pmirGLO-EGFR-wt; (g) mimic-piR-ace-1008436 and pmirGLO-EGFR-mut; and (h) mimic-NC and pmirGLO-EGFR-mut. Each group was transfected with 200 ng plasmid and 20 pmol mimic-piR (or mimic-NC). After transfection, cells were incubated at 37 °C for 24 h. Firefly and *Renilla* luciferase activities were measured using the dual-luciferase reporter assay system (Promega, Madison, WI, USA) and a dual-luciferase detection kit (Yeasen, Shanghai, China). Relative expression was calculated as the ratio of firefly luciferase to *Renilla* luciferase. The experiment was repeated in triplicate, and data are presented as means ± standard deviation (SD). Statistical analysis was performed using Student’s *t*-test with GraphPad Prism 8 software.

## 5. Conclusions

**Figure 9:**
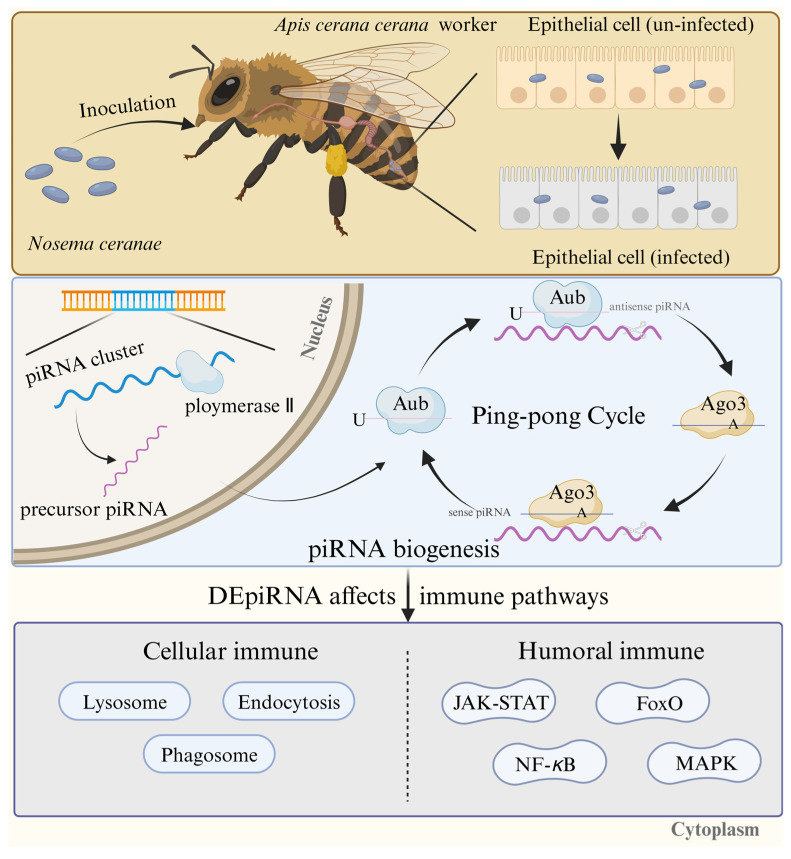
A hypothetical working model of the DEpiRNA-regulated response of *A. c. cerana* workers to *N. ceranae* infection. The yellow areas indicate changes in the intestinal epithelium after infestation of *A. c. ceranae* by *N. ceranae*, the blue areas identify the process of piRNA biosynthesis, and the grey areas indicate that DEpiRNA targets mRNAs and thus annotates in cellular and humoral immune-related pathways.

In conclusion, 539 piRNAs were identified in the midguts of *A. c. cerana* workers infected by *N. ceranae*. Two DEpiRNAs were detected in the midgut of workers at 7 dpi. The number of DEpiRNAs at 10 dpi increased to 46, among which, 35 DEpiRNAs were up-regulated. These results indicate that the number of DEpiRNAs in the midgut of workers escalates with the duration of infection by *N. ceranae*, and the expression patterns of piRNAs are altered. Furthermore, the DEpiRNAs influence the immune pathways of honeybees, thereby mediating the response to *N. ceranae* infection (Figure 9).

## Data Availability

All the data are contained within the article.

## Supplementary Materials

The following supporting information can be downloaded at: https://www.mdpi.com/article/10.3390/ijms26062402/s1.
